# The influence of total disc arthroplasty with Mobidisc prosthesis on lumbar spine and pelvic parameters: a prospective in vivo biomechanical study with a minimum 3 year of follow-up

**DOI:** 10.1186/s13018-022-03352-6

**Published:** 2022-10-15

**Authors:** Samir Smajic, Aleksandar Vujadinovic, Adnan Kasapovic, Dakheel A. Aldakheel, Yann Philippe Charles, Axel Walter, Jean-Paul Steib, Nicola Maffulli, Filippo Migliorini, Alice Baroncini

**Affiliations:** 1 Department of Orthopedic, Trauma and Spine Surgery, St. Josef Hospital, Linnich, Germany; 2 Department of Orthopedic and Trauma Surgery, Tuzla University Hospital, Tuzla, Bosnia and Herzegovina; 3 Department of Spine Surgery, Strasbourg University Hospital, Avenue Molière, 67200 Strasbourg, France; 4 Department of Orthopedic Surgery, Imam Abdulrahman Bin Faisal University of Dammam, Dammam, Kingdom of Saudi Arabia; 5 Department of Orthopedic and Trauma Surgery, University Hospital of Bonn, Bonn, Germany; 6 Department of Medicine, Surgery and Dentistry, University of Salerno, 84081 Baronissi, SA Italy; 7 Faculty of Medicine, School of Pharmacy and Bioengineering, Keele University, Stoke on Trent, ST4 7QB England UK; 8 Centre for Sports and Exercise Medicine, Barts and the London School of Medicine and Dentistry, Mile End Hospital, Queen Mary University of London, London, E1 4DG England UK; 9 Department of Orthopedic, Trauma, and Reconstructive Surgery, RWTH University Hospital, Pauwelsstraße 30, 52074 Aachen, Germany; 10 Department of Orthopaedic and Trauma Surgery, Eifelklinik St.Brigida, 52152 Simmerath, Germany

**Keywords:** Spine, Mobidisc, Spinopelvic alignment, Segmental lordosis

## Abstract

**Background:**

This study examined the impact of Mobidisc implant on spinopelvic parameters, with particular focus on the preservation of the lumbar lordosis (LL) and on the segmental lordosis (SL) of the treated and adjacent segments.

**Methods:**

A prospective study was conducted on 63 consecutive patients with symptomatic degenerative disc disease who underwent Mobidisc implantation at the Clinic for Spinal Diseases in Strasbourg, France. Based on the profile images of the whole, the following static spinopelvic parameters were measured and analysed: lumbar lordosis L1-S1 (LL), SL for L3-L4, L4-L5 and L5-S1, sacral slope (SS), pelvic tilt (PT) and pelvic incidence. In the lumbar spine images, the anterior (ADH) and posterior disc height (PDH) were measured prior to surgery and at the different follow-up appointments. The preoperative and postoperative values were compared and statistically analysed at different time intervals.

**Results:**

Sixty-three patients were included in the study. The average age of the patients was 41.4 years (range 27–59 years). The mean follow-up was 44 months (range 36–71 months). Overall, total disc replacement (TDR) led to an increase in LL which increased TED over time. The preoperative LL measured 48.9° ± 10.1° and 53.4° ± 9.9° at 3 years follow-up (*p* < 0.0001). In the cohort of patients who underwent TDR at L4-5, the LL increased from 51.6° ± 10° to 56.2° ± 9.2° at the last FU (*p* = 0.006). All other spinopelvic parameters remained stable between the preoperative values and the last follow-up. In the patients who underwent L5-S1 TDR, a significant increase in LL was also observed between preoperative data and at the last FU (from 47.8° ± 10.1° to 53.3° ± 10.1°, *p* < 0.0001). Following L5-S1 TDR, the SS increased from 32.9° ± 8.3° to 35.6° ± 7.4° (*p* = 0.05) and the PT decreased from 15.4° ± 6.2° to 11.6° ± 5.7° between preoperative values and the last follow-up. Considering the entire cohort, the SL L5-S1 increased significantly from 5.9° ± 4.2° preoperatively to 8.1° ± 4.4° (*p* < 0.01) at the last FU, while at the L4-L5 level, the SL remained stable from 9.9 ± 4.5° to 10.7° ± 3.8° (*p* = 0.3). After L4-5 TDR, an increase in ADH and PDH at the treated level was observed, while these parameters progressively decreased in the adjacent segment. In patients who underwent L5-S1 TDR, a significant increase in L5-S1 ADH and PDH was observed from 18.8 ± 9.1 to 28.4 ± 11.1 and from 9.5 ± 3.8 to 17.6 ± 9.5 pixels, respectively. ADH and PDH at the proximal adjacent levels L3-4 and L4-5 were reduced. We did not observe any case of implant failure or damage to the bone/implant interface.

**Conclusion:**

TDR with Mobidisc allows for an improvement of LL and SL at the treated level. An increase in both anterior and posterior disc height was observed at the treated level. While disc height decreased at the adjacent level, further studies are required to investigate whether these changes are clinically relevant.

## Introduction

Chronic lumbar pain syndrome caused by degeneration of the intervertebral disc is a leading cause of disability in the working population worldwide [1]. Given the associated high burden of medical care and the loss of working time, chronic lumbar pain represents a major socioeconomic issue [2, 3]. The first line management of chronic lumbar pain consists of physical therapy and drugs administration [4]; surgery is considered only when these measures do not provide sufficient pain relief. Spinal fusion represents the gold standard for the surgical management of degenerative disc disease (DDD) [5]. However, this procedure leads to a loss of spine mobility and, in turn, to a possible degeneration of the adjacent segments [6–8].

Numerous efforts have focussed on total disc replacement (TDR), or total disc arthroplasty (TDA), as an alternative to avoid fusion [9]. The first study on TDR in the USA was conducted in March 2000 as part of a study approved by the US National Food and Drug Administration [10]. Over time, numerous investigations assessed the safety and efficacy of TDR, with positive outcomes in the mid-to-long term [11, 12]. Recently, a second-generation lumbar prosthesis with mobility control in all directions has been developed (Mobidisc, Zimmer Biomet Spine, 80,021 Westminster, US). Its self-centring mobile insert enables the maintenance of a constant centre of rotation, both in translation (forward–backward) and rotation, thus replicating the physiological mobility of the intervertebral disc. This study examined the impact of Mobidisc implant on the spinopelvic parameters, with particular focus on the preservation of the lumbar lordosis (LL) and on the segmental lordosis (SL) of the treated and adjacent segments.

## Materials and methods

### Ethics

The present study was performed according to the Strengthening the Reporting of Observational Studies in Epidemiology (STROBE). The present study was approved and registered by the ethics committee of the University of Tuzla, Bosnia (project ID 02-09/2-99-1/21) and conducted according to the principles expressed in the Declaration of Helsinki. All patients were able to understand the nature of their treatments and provided written consent to use their data for research purposes.

### Patient selection

A prospective study was conducted on all consecutive patients with symptomatic DDD who underwent Mobidisc implantation at the Department of Spine Surgery at University of Tuzla, Bosnia and at the Clinic for Spinal Diseases in Strasbourg, France. Inclusion criteria were age < 65 years, pain in the lumbosacral spine caused by DDD as documented by preoperative imaging, and availability of pre- and postoperative radiographs. Patients with predominant radicular pain, neurological deficits, documented osteoarthritis of the facet joints, osteoporosis, scoliosis, vertebral fracture, infection or previous spine surgery were excluded from the analysis. Mobidisc implantation at more than one level also led to exclusion from the study.

### Outcomes of interest

Plain standing radiographs of the lumbar and whole spine in anteroposterior and lateral projections were obtained for all patients before surgery and 6 weeks, 3, 6, 12 and 36 months, after the operation. The same protocol was used to obtain all images. All radiographs were scanned using Vidar Corporation's digitizer and transferred to Spine Vision software^®^, which was used to measure the desired parameters. One author, an experienced spine surgery consultant, performed all the measurements.

Based on the profile images of the whole, the following static spinopelvic parameters were measured and analysed: lumbar lordosis L1-S1 (LL), segmental lordosis (SL) for L3-L4, L4-L5 and L5-S1, sacral slope (SS), pelvic tilt (PT) and pelvic incidence (PI). In the lumbar spine images, the anterior (ADH) and posterior disc height (PDH) were measured prior to surgery and at the different follow-ups. The preoperative and postoperative values were compared and statistically analysed at different time intervals.

### Statistical analysis

In statistical data processing, standard methods of descriptive and inferential statistics were used. To test statistical significance among the samples, parametric and nonparametric significance tests (*t* test, Chi2 test, Mann–Whitney test, F-test, An analysis) were used, as well as correlation analysis, and testing of its significance. Statistical hypotheses were tested at a significance level of *p* < 0.05. All the statistical analyses were performed using SPSS Statistics 17.0 and Statistics 8.0.

## Results

### Demographics

During the observation period, 80 patients with symptomatic DDD underwent surgery at our institution. Nine patients were excluded because they had had previous fusion surgery. One further patient was excluded because TDR was performed at three levels. Seven patients were excluded because of incomplete documentation. Thus, 63 patients were included in the study: 18 males (28.6%) and 45 females (71.4%), a male and female ratio of 1:2.5. The average age of the patients was 41.4 years (range 27–59 years). The mean follow-up was 44 months (range 36–71 months). Forty-four subjects (69.8%) underwent L5-S1 TDR, and 19 (30.2%) received L4-L5 TDR. Patients’ demographics are summarized in Tables 1 and 2.

**Table 1:** summary of the patients’ distribution by gender and treated level

		Operated level		Tot
		L5/S1	L4/L5	
Gender	*M*	6 (9.52%)	12 (19.05%)	18 (28.57%)
	*F*	38 (60.32%)	7 (11.11%)	45 (71.43%)
Tot		44 (69.84%)	19 (30.16%)	63 (100.00%)

**Table 2:** Age distribution of the included patients

Age	*M *(*n* = 18)		*F* (*n* = 45)	
	*N*	%	*N*	%
20–30	0	0.00	3	6.67
31–40	4	22.22	20	44.44
41–50	11	61.11	19	42.22
51–60	3	16.67	3	6.67

### Radiographic results

#### Spinopelvic parameters

Overall, TDR led to an increase in LL which increased TED over time. The preoperative LL measured 48.9° ± 10.1° and increased to 53.4° ± 9.9° at 3 years follow-up (*p* < 0.0001). Over time, the SS increased from 34° ± 7.7° to 36.5° ± 6.9° at the last FU (*p* < 0.0001), and PT decreased from 15.3° ± 6.6° to 12.71 ± 6.8° at the last FU (*p* = 0.001). The PI, as expected, remained unchanged over time (47.4° ± 8.7°).

In the cohort of patients who underwent TDR at L4-5, the LL increased from 51.6° ± 10° to 56.2° ± 9.2° at the last FU (*p* = 0.006). All other spinopelvic parameters remained stable from the preoperative values and the last follow-up (SS from 36.5° to 38.5°, *p* = 0.1; PI from 15.0° to 15.2°, *p* = 0.9; PI from 51.9° to 51.1°, *p* = 0.6). In the patients who underwent L5-S1 TDR, a significant increase in LL was also observed between preoperative data and at the last FU (from 47.8° ± 10.1° to 53.3° ± 10.1°, *p* < 0.0001).

Following L5-S1 TDR, the SS increased from 32.9° ± 8.3° to 35.6° ± 7.4° (*p* = 0.05) and the PT decreased from 15.4° ± 6.2° to 11.6° ± 5.7° between preoperative values and the last follow-up. PI remained stable.

#### Segmental lordosis

Considering the entire cohort, the SL L5-S1 increased significantly from 5.9° ± 4.2° preoperatively to 8.1° ± 4.4° (*p* < 0.01) at the last FU, while the SL at the L4-L5 level remained stable (9.9 ± 4.5° to 10.7° ± 3.8°; *p* = 0.255). Surgery did also not affect the SL of the proximal adjacent segment L3-4, which remained stable (6.36° ± 0.3° to 6.01° ± 0.4°). In the group of patients with L4-L5 TDR, the SL at the instrumented segment increased from 9.2° ± 4.3° to 12.2° ± 4.1° (*p* = 0.03), while no significant changes were observed in the SL L3-4 and L5-S1. Considering patients who underwent L5-S1 TDR, the SL increased from 5° ± 3.4° to 8.8° ± 4.5° (*p* < 0.0001). No significant changes in the L3-4 and L4-5 discs were observed.

#### Disc height

After L4-5 TDR, an increase in ADH and PDH at the treated level was observed, while these parameters progressively decreased in the adjacent segment. Anterior disc height at the L4-L5 level increased from preoperative 23.81 to postoperative 34.15 pixels (*p* < 0.05), while posterior disc height increased, from preoperative 10.68 to postoperative 14.77 pixels (*p* < 0.01).

The values of ADH and PDH at disc height values at adjacent levels, L3-L4 and L5-S1, decreased over time despite a postoperative increase. At L3/4, ADH (Table 3). Only the PDH L5-S1 remained stable at the last follow-up.

**Table 3:** Preoperative and postoperative values of anterior and posterior disc height at L3-L4 and L5-S1 after L4-5 TDR

	ADH L3-L4 after TDR L4-5			PDH L3-4 after TDR L4-5			ADH L5-S1 after TDR L4-5			PDH L5-S1 after TDR L4-5		
	Baseline	6 weeks	3 years	Baseline	6 weeks	3 years	Baseline	6 weeks	3 years	Baseline	6 weeks	3 years
Mean (pixel)	10.6	26.4	14.7	24.7	30.2	13	30.4	32.9	18.1	14.9	25.1	10.6
SD	3.4	24.8	5.6	6.3	24.6	3.2	13.6	18.4	8.1	7.7	18.9	5.5
*p*	1	0.5	0.005	1	0.3	< 0.0001	1	0.5	0.003	1	0.03	0.08

In patients who underwent L5-S1 TDR, a significant increase in L5-S1 ADH and PDH was observed from 18.8 ± 9.1 to 28.4 ± 11.1 and from 9.5 ± 3.8 to 17.6 ± 9.5 pixels, respectively. ADH and PDH at the proximal adjacent levels L3-4 and L4-5 were reduced (Table 4).

**Table 4:** Preoperative and postoperative values of anterior and posterior disc height at L3-L4 and L4-6 after L5-S1 TDR

	ADH L4-5 after TDR L5-S1			PDH L4-5 after TDR L5-S1			ADH L3-4 after TDR L5-S1			PDH L3-4 after TDR L5-S1		
	Baseline	6 weeks	3 years	Baseline	6 weeks	3 years	Baseline	6 weeks	3 years	Baseline	6 weeks	3 years
Mean (pixel)	39.9	37.1	27.2	20.3	19.9	14.6	34.9	30.8	22.3	22.5	20.4	14.8
SD	14.3	11.1	12.2	8.1	7.1	6.4	12.8	10.1	9.8	8.2	7.1	5.6
*p*	1	0.1	< 0.0001	1	0.7	< 0.0001	1	0.01	< 0.0001	1	0.05	< 0.0001

### Complications

In the present study, we did not observe any case of implant failure or damage to the bone/implant interface. In three cases of L4-L5 TDR, a laceration of the iliac vein occurred. One patient presented retrograde ejaculation after L5-S1 TDR. One case of intestinal intussusception occurred, which required surgical therapy.

## Discussion

The main findings of this study are that, following TDR, there was an increase both in LL and in the SL of the operated segments. The other spinopelvic parameters remained stable. An increase in ADH and PDH was observed at the treated level, while ADH and PDH decreased at the adjacent levels.

Maintaining physiological lordosis is an essential goal in any reconstructive procedure on the lumbar spine [13]. While none of the patients involved in our study presented a preoperative loss of lordosis which required correction, we observed an improvement in LL in all the operated patients. In the observed cohort, the LL at the last follow-up measured 53.43°: These results compare well with the data available in the literature, as Huang et al. observed an average postoperative LL of 58.6° [13] and Tropiano et al. of 61.9° [14]. Tournier et al. analysed the postoperative LL in three types of disc replacement implants: the discs-Prodisc L^®^ (Synthes Spine, West Chester, NY,USA), Maverick^®^ (Medtronic Sofamor Danek, Inc., Mem-phis, TN, USA), and SB Charite III^®^ (DePuy spine, Rayn-ham, MA, USA). The authors evidenced an increase in the LL from 50.3° to 52.4° [15]. Similar results were obtained by Chang and colleagues, who observed a mean increase in LL from 30.5° to 40.8° [16].

In the present study, SL increased significantly only at the L5/S1 level: An excessive increase in the SL may lead to an impingement of the posterior structures [17]. This phenomenon occurred when the SL angle measured more than 16° with SB Charite III implants [17]. In our cohort, four L4/5 patients and three L5/S1 patients presented a SL greater than 16° after TDR. Considering that the prosthesis analysed in the present work did not allow to obtain such SL angles, and given that no cases of implant damage was observed, we may infer that the use of Mobidisc Prosthesis does not appear to cause impingement of the posterior structures in the short-to-mid term. A longer follow-up will be required to investigate the long-term effects of the increase in SL in patients who underwent TDR with the Mobidisc implant.

The Mobidisc prothesis allowed to increase PDH and thus leads to indirect decompression of the neural structures. The process of disc degeneration leads to a loss of water content and anatomic disruption of the affected level, which results in a reduction in disc height [18]. This, in turn, results in an increased load on the facet joints, followed by the development of OA and the onset of pain [19]. Restoring disc height thus aims not only to indirectly decompress, but also to restore the physiological load distribution and, consequently, to preserve the facet joints. However, oversizing the implant may cause overextension of the posterior longitudinal ligament and muscular structures potentially leading to pain and muscle fatigue. Thus, particular care should be put into sizing of implant. TDR with Mobidisc did not allow to restore the disc height levels observed in asymptomatic subjects (L4/5 ADH: 10.8 mm, L4/5 PDH: 7.2 mm; L5/S1 ADH 10.4 mm, L5/S1 PDH 6 mm) [20]. As no revision for radicular pain or articular degeneration was required within the 3-years follow-up, the increase in disc height obtained with TDR allows sufficient neural decompression and physiological load sharing. Further biomechanical studies and a longer follow-up will be required to further investigate this aspect. In our cohort, a decrease in disc height at the levels adjacent to the TDR was observed: To the best or our knowledge, this is the only study to report this finding. While for the aforementioned reasons, a decrease in disc height may result in degeneration of the adjacent segment, this complication was not reported during the 3-years observation period. A longer follow-up with clinical and imaging data will be required to ascertain whether this finding has clinical relevance.

In the present study, the increase in lumbar lordosis was accompanied by slight changes in the sacral slope and pelvic tilt. These changes are expected, given the closed interactions between the spinopelvic parameters; however, it is questionable whether the modifications of SS and PT, despite being statistically significant, bear any clinical relevance. None of the included patients showed radiographic signs of sagittal malalignment, so that an improvement in PT was not necessary. These considerations are coherent with the finding of other study groups, which did not report any significant change in the spinopelvic parameters despite an increase in LL [16, 21].

Regarding the rate of complications, the literature reports a complication range of 10–20% [14, 21]. Tropiano et al. observed a 6% reoperation rate after implantation of a ProDisc II or SB Charite III prosthesis [14]. The most common causes of revision were vertebral fracture, wrong implant positioning and persistent radiculopathy [14]. In a further study with a follow-up of almost 9 years, Tropiano et al. followed 64 patients after implantation of a ProDisc prosthesis. Complications occurred in five patients (9%): one deep venous thrombosis, one laceration of the iliac vein (which was primarily repaired), one transient episode of retrograde ejaculation and two hernias at the incision site [22]. No complications caused by prosthesis damage were observed. Zigler et al. observed one case of dislocation of the polyethylene insert, one vein laceration, one superficial infection, and two cases of radicular pain after ProDisc TDR [23]. Another long-term study observed prosthesis displacement in 3/106 patients, and in two cases subluxation of the mobile insert [24]. No prosthesis damage was reported in our study, nor were there any other signs of biomechanical damage.

## Conclusion

TDR with Mobidisc allows for an improvement of LL and SL at the treated level. An increase in both anterior and posterior disc height was observed at the treated level. While disc height decreased at the adjacent level, further studies are required to investigate whether these changes are clinically relevant.

## Data Availability

The data underlying this article are available in the article and in its online supplementary material.

## Abbreviations

LL: Lumbar lordosis
SL: Segmental lordosis
SS: Sacral slope
PT: Pelvic tilt
PI: Pelvic incidence
ADH: Anterior
PDH: Posterior disc height
DDD: Degenerative disc disease
TDR: Total disc replacement
TDA: Total disc arthroplasty
